# Screening tools for obstructive sleep apnea in patients with systemic lupus erythematosus

**DOI:** 10.1186/s42358-025-00503-1

**Published:** 2025-11-29

**Authors:** Lia Belchior Mendes Bezerra, Manoel Alves Sobreira Neto, Juliana Arcanjo Lino, Adriana Lima Araújo, Carlos Ewerton Maia Rodrigues

**Affiliations:** 1 Graduate Program in Medical Sciences, University of Fortaleza (Unifor), Washington Soares Avenue, 1321, Edson Queiroz, Fortaleza, 60811-905 Brazil; 2 Department of Internal Medicine, Federal University of Ceara, Fortaleza, Brazil; 3 Department of Physiology and Pharmacology, Federal University of Ceara, Fortaleza, Brazil

**Keywords:** Obstructive sleep apnea, Systemic lupus erythematosus, Sleep apnea questionnaires, Screening

## Abstract

**Background:**

Obstructive sleep apnea (OSA) seems to be an important comorbidity in systemic lupus erythematosus (SLE) but studies on prevalence have inconsistent results. Moreover, uncertainty exists as to the reliability of questionnaires that could predict who should be considered for sleep testing. We aimed to determine the prevalence OSA in SLE patients and in healthy controls, and to evaluate the performance of two widely used OSA screening tools (STOP-Bang; NoSAS).

**Methods:**

Selected patients underwent a home sleep apnea testing (HSAT), or type III polysomnography, using a portable ApneaLink^TM^ Air device (ResMed^®^), Martinsried, Germany. OSA was defined as apnea-hypopnea index ≥5 events/h. The probability of OSA was evaluated with two questionnaires: STOP-Bang and NoSAS. The ability of these questionnaries to diagnose apnea in SLE patients was analyzed with ROC curves, determining the area under the ROC curve (AUC-ROC) and the respective 95% confidence intervals.

**Results:**

The final sample included 19 SLE patients (women: 89.5%; mean age: 35.6 ± 10.3 years; mean BMI: 27.87 ± 6.87 kg/m^2^) and 20 controls. The prevalence of OSA was 26.4% for SLE patients and 35% for controls (*p* = 0.360). OSA patients had higher BMI (*p* = 0.043). STOP-Bang scores were higher for patients with OSA than for patients without OSA (3 [2; 3] vs. 1 [0; 2], *p* = 0.001). The same was observed for NoSAS scores (7 [7; 9] vs. 2 [0; 3], *p* < 0.005). The area under the ROC curve (AUC) was greater for NoSAS than for STOP-Bang (0.957, 95%CI: 0.710–1.000, *p* = 0.003) vs. (0.914, 95%CI 0.786–1.000, *p* = 0.003), respectively.

**Conclusion:**

SLE patients and controls had a similar frequency of OSA in this study. Both OSA screening tools (NoSAS and STOP-Bang scores) detected OSA with reasonable accuracy in this population but at cut-offs lower than the usual.

## Introduction

Systemic lupus erythematosus (SLE) is a chronic autoimmune disorder involving autoantibody formation and immune complex deposition [1]. SLE can affect several organs, with alternating periods of activity and remission. Clinical manifestations are diverse and can be severe, with high levels of morbidity and mortality. Even when properly treated, mortality is 2–3 times higher in SLE patients than in the general population, mostly due to renal, cardiovascular, and infectious complications [2]. The negative impact of SLE may be attenuated with regular physical exercise, good quality sleep, and preventive mental health care, among other lifestyle measures [3].

Obstructive sleep apnea (OSA) manifests as recurrent episodes of partial obstruction (hypopnea) or complete obstruction (apnea) of the upper airway during sleep leading to intermittent hypoxia, transient hypercapnia, and sleep fragmentation. It can occur with or without symptoms. Snoring, excessive sleepiness, fragmented sleep and fatigue upon waking are some of the most common complaints [4]. The prevalence of OSA is on the rise worldwide (16.4–36.1%) [5–7], possibly in association with populational aging and the global obesity epidemic. In Brazil, the prevalence is estimated at 32.9% [6]. Besides sleep-related symptoms, OSA has been associated with an increased risk of cardiovascular and cerebrovascular diseases and it have been identified as an independent risk factor for hypertension, stroke, myocardial infarction and mortality. Several pathophysiological factors contribute to the relationship between OSA and cardiovascular risk, including neuro-hormonal dysregulation, endothelial dysfunction, and inflammation [5]. Sleep disturbances may occur in several rheumatological disorders, such as rheumatoid arthritis and SLE and this fact can amplify the cardiovascular morbidity inherent in these diseases [5]. Animal models have shown that these conditions may be associated with sleep abnormalities, but the physiopathological mechanisms involved remain unclear [8, 9]. A possible explanation linking OSA and autoimmune diseases may involve chronic inflammation caused by repeated episodes of interrupted breathing and oxygen desaturation during sleep, as well as the role of intermittent hypoxemia and its association with endothelial dysfunction [9–11]. Apneas causing chronic intermittent hypoxia may activate an inflammatory cascade resulting in elevated cytokine levels possibly contributing to autoimmunity.[10, 11]

SLE patients report more sleep disorders than the general population [12]. According to one study, SLE patients are 2.5 times more likely to have poor sleep quality than patients with other chronic illnesses [13]. Many believe components intrinsic to SLE can cause such changes, disturbing the relationship between sleep and immunity [8]. Symptoms of depression, pain and disease activity are strongly associated with poor sleep quality [14].

OSA in SLE manifests primarily as poor sleep quality, daytime sleepiness, and chronic fatigue [13–15], but studies on prevalence have yielded inconsistent results (26–68%) [9, 15–17].

Little has been published on the prevalence of OSA in SLE and on how the association affects the development of SLE. In this study we therefore set out to determine the prevalence of OAS in a sample of SLE patients vs. controls, and to evaluate the performance of OSA screening tools in this population.

## Methods

### Study approval

This cross-sectional observational study was conducted at an outpatient service at a referral hospital in Northeastern Brazil (Hospital Geral de Fortaleza) between September 2022 and October 2023.

In the first stage of the study, we compared type III polysomnography (PSG) findings for SLE patients vs. body mass index (BMI), sex and age-matched controls from the local community. In the second stage we evaluated the performance of two OSA screening tools (Snoring, Tired, Observed apneas, Pressure, BMI, Age, Neck size, Gender (STOP-Bang) and NoSAS) in our sample of SLE patients.

This research project was approved by the hospital’s ethics committee via *Plataforma Brazil* (CAAE No. 63086722.7.0000.5052). Informed written consent was provided by all participants and/or their caretakers. The study protocol complied with the guidelines of the Declaration of Helsinki.

### Patients

Adult patients ( > 18 years), both sexes, diagnosed with SLE according to the criteria of the American College of Rheumatology revised by the European League Against Rheumatism (ACR/EULAR 2019) [18, 19] followed up regularly at the rheumatology outpatient service of a tertiary general hospital were approached consecutively during routine check-ups and invited by the attending physician to participate in the study. The recruitment took place from June 2022 to October 2023. The exclusion criteria were: history of other concomitant autoimmune rheumatic disorders (i.e., Sjögren’s disease, rheumatoid arthritis) and fibromyalgia, known non-respiratory sleep disturbances, known neuromuscular disease, high risk of sleep-related alveolar hypoventilation [7], use of psychotropic/opioid/sedative/hypnotic medication, clinically significant heart failure or echocardiogram showing ejection fraction < 45% or signs of congestive heart failure, pregnancy or breastfeeding, chronic kidney failure (defined as creatinine clearance < 30 mL/min/1.73 m^2^) or nephrotic syndrome (defined as proteinuria > 3.5 g/1.73 m^2^/day, hypoalbuminemia < 3.0 g/dL, and edema) [7, 20].

The selected SLE patients were given 4 questionnaires: a general questionnaire on demographics and clinical history, two OSA screening tools (STOP-BANG and NoSAS), and the Epworth sleepiness scale (ESS). Finally, all participants had their height (m), weight (kg), BMI (kg/m^2^) and neck circumference (cm) (just below the laryngeal prominence, below the hyoid bone, along the larynx) measured, followed by the scheduling of at-home polysomnography [21]. Traditionally, OSA diagnosis is made through full polysomnography (PSG) conducted in a sleep laboratory. However, this method has significant practical limitations, such as high cost, limited availability of specialized centers, and the need to transport the patient away from their home and their usual sleeping conditions. The AASM Practice Guideline for Diagnostic Testing for OSA indicates a strong correlation between the Home Sleep Apnea Test (HSAT) and PSG indices. Considering these limitations, the HSAT has proven to be a safe, logistically feasible, and cost-effective alternative for patients [7].

The controls consisted of BMI, age, and sex-matched healthy volunteers from the community, at a proportion of 1:1. Patients and controls were compared regarding OSA frequency based on the apnea-hypopnea index (AHI).

### Data collection

Information on medical history and the parameters required to individually score the Systemic Lupus Erythematous Disease Activity Index (SLEDAI-2k) [22] and the Systemic Lupus International Collaborating Clinics/American College of Rheumatology (SLICC/ACR) Damage Index (DI) [23] were retrieved from the patients’ records.

The SLEDAI-2k measures the overall disease activity in the preceding 10 days and is a predictor of mortality. It uses 24 descriptors of clinical manifestations and lab tests, encompassing 9 organ systems, and yields a score of up to 105. The most adequate cut-off to define active disease and the need for therapy adjustment is 3 or 4, but trend analysis is just as important in decision making [22].

The SLICC-DI measures the level of sequelae and irreversible cumulative damage from SLE and/or therapy for SLE, using a scale from 0 to 44 and covering 12 potentially affected systems [23].

Other clinical data on medications, cumulative dose of corticoids, and lab findings were retrieved from the patients’ electronic records.

The patients were submitted to HSAT, or type III polysomnography, using a portable ApneaLink^TM^ Air device (ResMed^®^), Martinsried, Germany. The test monitored respiratory effort with a single thoracoabdominal piezo belt, pulse rate and peripheral oxygen saturation (SpO_2_), and nasal flow and snoring using a disposable nasal cannula. Since the test did not capture EEG data, no information on sleep stages or architecture was generated [7, 24]. The test was administered in the home setting up to one month after the first interview at the outpatient service.

The quality of the exam was evaluated, and respiratory events were quantified manually by sleep specialist aided by the software Airview^®^, following the HSAT guidelines for adults in the AASM manual for the scoring of sleep and associated events (v. 2.6) [25]. Apnea was defined as ≥90% reduction in the amplitude of the flow curve in relation to baseline, while hypopnea was defined as a ≥ 30% reduction in the amplitude of the flow curve associated with ≥3 points of oxyhemoglobin desaturation, both of which lasting 10 seconds or more. In addition, apnea was classified as central or obstructive depending on whether respiratory efforts were absent or persistent during the events, respectively [26]. Finally, the sum of apnea/hypopnea events per hour of monitoring was used to determine the AHI. We also registered the total time spent with oxygenation < 90% (T90%), minimum peripheral oxygen saturation (SpO_2_), and the oxygen desaturation index (ODI), defined as the number of episodes of ≥3% reduction in oxyhemoglobin levels per hour in relation to baseline.

A technically acceptable test had to include at least 4 hours of adequate oximetry and flow data obtained in a single recording during the habitual sleep period. Tests not meeting the quality criteria were excluded from the analysis of OSA prevalence.

OSA was considered present when AHI was ≥5 (i.e., 5 or more events per hour) with symptoms. Three levels of severity were considered: mild (5–14.9 events/h), moderate (15–29.9 events/h), and severe (≥30 events/h) [27].

SLE patients and controls performed the HSAT under similar conditions.

Daytime sleepiness was evaluated with the Epworth Sleepiness Scale (ESS) questionnaire which contains 8 habitual situations in which the probability of falling asleep is rated from 0 (none) to 3 (always). A final score in the 10–24 range indicates excessive daytime sleepiness [28].

The probability of OSA was evaluated with two questionnaires: STOP-Bang and NoSAS. The former contains 8 dichotomous items (yes/no): snoring, tiredness, observed apnea and high blood pressure (STOP), and BMI, age, neck circumference and male gender (Bang). Scores range from 0 to 8, with ≥5 as cut-off for high risk of OSA [7, 19, 27]. The latter features 5 items, yielding a score between 0 and 17, broken up into neck circumference > 40 cm (4 points), BMI 25–30 kg/m^2^ (3 points) or ≥ 30 kg/m^2^ (5 points), snoring (2 points), age > 55 years (4 points), and male gender (2 points). The usual cut-off for high risk of OSA is ≥ 8 [29, 30].

## Statistical analysis

Categorical variables were expressed as absolute numbers and percentages. The chi-square test or Fisher’s exact test was used to verify associations between categorical variables (as applicable). Continuous variables were submitted to the Shapiro-Wilk test of normality of distribution, then analyzed using histograms, Q-Q plots, and dispersion measures. Parametric data were expressed as mean value ± SD, while non-parametric data were expressed as median and interquartile range. Pairwise comparisons were performed with Student’s *t* test for parametric data and the Mann-Whitney U test for non-parametric data. Correlations between continuous variables were evaluated with Spearman correlation coefficients.

The ability of STOP-BANG and NoSAS to diagnose apnea in SLE patients was analyzed with receiver operating characteristic (ROC) curves, determining the area under the ROC curve (AUC-ROC) and the respective 95% confidence intervals (CI). In addition, the best cut-off was calculated with the Youden index (sensitivity + specificity − 1). We conducted additional analyses to estimate the effect size. We calculated Cohen’s d for quantitative variables and used Cohen’s w for categorical variables to assess the strength of associations. Furthermore, we evaluated the statistical power for each comparison, based on the effect size of each association. All statistical analyses were performed using the software SPSS for Macintosh (v. 23.0, Armonk, NY: IBM Corp), with the level of statistical significance set at 5% (*p* < 0.05).

## Results

### Selection of participants

**Fig. 1:**
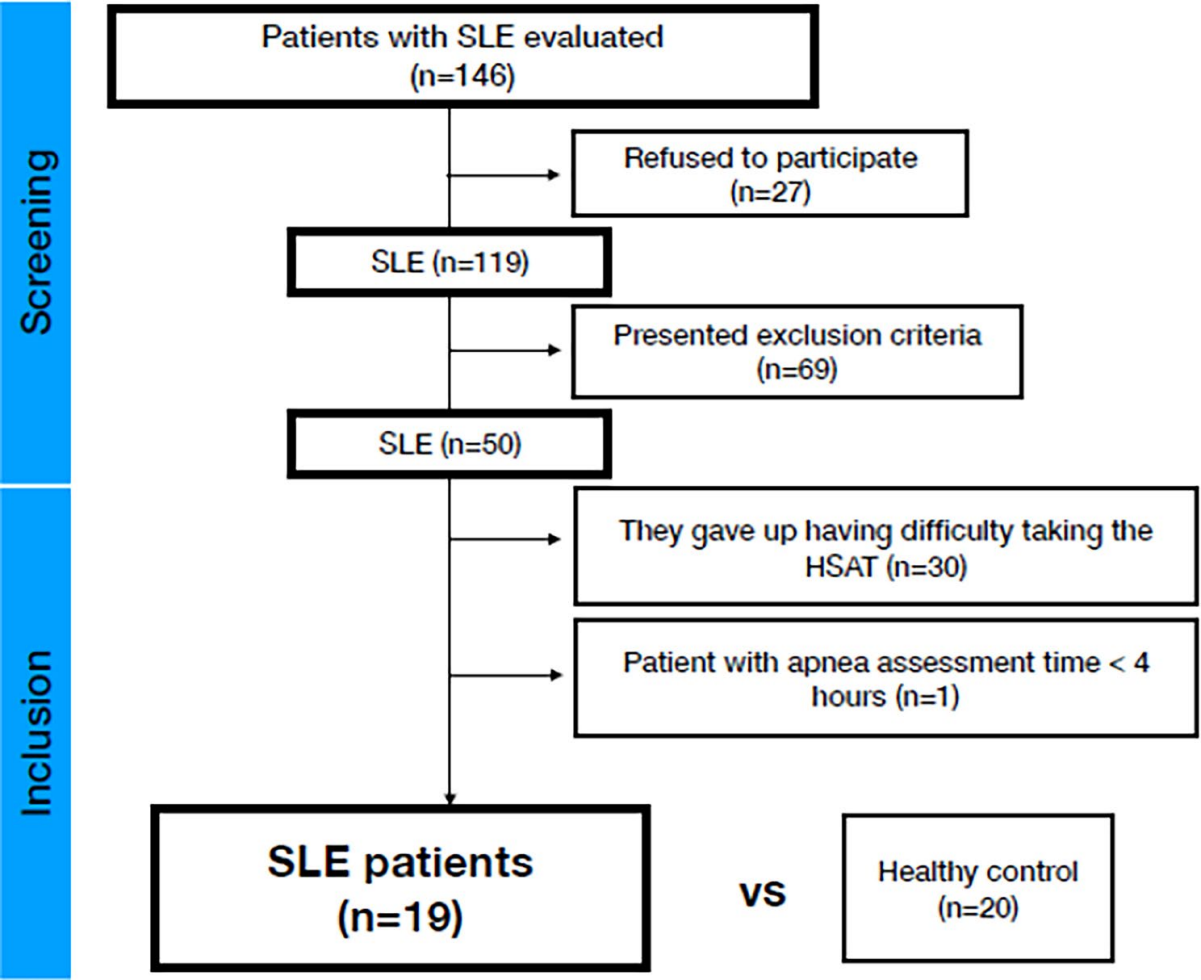
Flowchart of evaluated SLE patients. SLE: systemic lupus erythematosus, HSAT: home sleep apnea testing

During the study period, 627 patients were seen at the rheumatology service, 146 of whom were invited to participate. One or more exclusion criteria were applicable to 69 and 27 declined participations, yielding an initial sample of 50 participants. Subsequently, 30 patients were excluded from the study due to difficulties in carrying out the HSAT (difficulty with transportation to pick up and return the polygraphy equipment, shared bed with children, very distant housing location, and fear that the equipment would cause harm), leaving a final sample of 20 patients. A single test was eventually excluded due to insufficient time of monitoring (Fig. 1).

To proceed with the OSA frequency evaluation, 20 healthy controls matched for sex (*p* = 1.00), age (*p* = 0.748) and BMI (*p* = 0.661) were recruited from the local community (Table 1).

**Table 1:** Clinical-demographical profile vs. Apnea-Hypopnea Index (AHI) values of SLE patients

	**SLE** (*n* = 19)	**AHI** (events/hour)		*p***-value**	**Effect size (CI 95% for Cohen’s d)**^**1**^	Statistical Power
		** < 5** (*n* = 14)	**≥ 5** (*n* = 5)			
**Sex**				0.4	0.0	5%
Female	17 (89.5)	13 (92.9)	4 (80)			
Male	2 (10.5)	1 (7.1)	1 (20)			
**Age** (years)	35.6 ± 10.3	35 ± 8.4	37.2 ± 15.6	0.7	−0.2 (−1.3; 0.8)	6.6%
**Race**				0.2	0.4	44%
White	5 (26.3)	5 (35.7)	0 (0)			
Mixed brown race	13 (68.4)	8 (57.1)	5 (100)			
Black	1 (5.3)	1 (7.1)	0 (0)			
**BMI** (Kg/m^2^)	27.9 ± 6.9	26 ± 4.9	33.1 ± 9.2	**0.0**	**-1.1 (−2.3; 0.0)**	**54%**
**BMI (classification)**				**0.0**	**0.5**	**68%**
Eutrophic	7 (36.8)	7 (50)	0 (0)			
Overweight	8 (42.1)	6 (43)	2 (40)			
Obese	4 (21.1)	1 (7)	3 (60)			
**Menopausal**				0.3	0.2	12.8%
No	13 (68.4)	11 (78.6)	2 (40)			
Yes	4 (21.1)	2 (14.3)	2 (40)			
Not applicable	2 (10.5)	1 (7.1)	1 (20)			
**Arterial hypertension**				1	0.0	5%
No	14 (73.7)	10 (71.4)	4 (80)			
Yes	5 (26.3)	4 (28.6)	1 (20)			
**Diabetes**				0.2	0.1	8.7%
No	18 (94.7)	14 (100)	4 (80)			
Yes	1 (5.3)	0 (0)	1 (20)			
**Physical activity**				0.1	0.3	29.6%
No	10 (55.6)	9 (69.2)	1 (20)			
Yes	8 (44.4)	4 (30.8)	4 (80)			
**Smoking**				1	0.0	5%
No	18 (94.7)	13 (92.9)	5 (100)			
Yes	1 (5.3)	1 (7.1)	0 (0)			
**Alcohol use**				-	-	-
No	19 (100)	14 (100)	5 (100)			
Yes	0 (0)	0 (0)	0 (0)			
**Tiredness**				0.3	0.2	15.2%
No	9 (47.4)	8 (57.1)	1 (20)			
Yes	10 (52.6)	6 (42.9)	4 (80)			
**Snoring**				0.3	0.2	15.2%
No	9 (47.4)	8 (57.1)	1 (20)			
Yes	10 (52.6)	6 (42.9)	4 (80)			
**Good sleep quality**				0.6	0.1	7%
No	8 (42.1)	5 (35.7)	3 (60)			
Yes	11 (57.9)	9 (64.3)	2 (40)			
**Time of disease** (months)	60 (11; 168)	46 (6; 180)	72 (60; 108)	0.6	0.2 (−0.9; 1.2)	6%
**SLEDAI 2K**	0 (0; 4)	0 (0; 2)	0 (0; 4)	0.9	−0.2 (−1.3; 0.8)	7.1%
**SLICC**				1	0.0	5%
0	13 (68.4)	10 (71.4)	3 (60)			
1	6 (31.6)	4 (28.6)	2 (40)			
**Cumulative dose of corticoids**	2.8 (0.5; 11.3)	5 (1.86; 12.5)	0.5 (0; 0.95)	0.1	0.7 (−0.3; 1.9)	28.4%
**Current corticoid use**				1	0.0	5%
No	14 (73.7)	10 (71.4)	4 (80)			
Yes	5 (26.3)	4 (28.6)	1 (20)			
**Hydroxychloroquine**				-	-	-
No	0 (0)	0 (0)	0 (0)			
Yes	19 (100)	14 (100)	5 (100)			
**Mycophenolate**				1	0.0	5%
No	13 (68.4)	10 (71.4)	3 (60)			
Yes	6 (31.6)	4 (28.6)	2 (40)			
**Methotrexate**				0.2	0.1	8.7%
No	18 (94.7)	14 (100)	4 (80)			
Yes	1 (5.3)	0 (0)	1 (20)			

Abbreviations: AIH – apnea-hypopnea index, SLE – Systemic lupus erythematosus, BMI – body mass index, SLEDAI 2k – Systemic Lupus Erythematous Disease Activity Index 2000, SLICC-DI – Systemic Lupus International Collaborating Clinics/American College of Rheumatology Damage Index

Categorical variables expressed as absolute numbers and percentages (in parentheses). Quantitative variables expressed as mean value ± standard deviation, or median and interquartile range (in parentheses).

^1^ Cohen’s *d* used for continuous data, and Cohen’s *w* for categorical data.

### Clinical-epidemiological aspects of SLE patients

Most SLE patients were women of childbearing age (mean age: 35.6 ± 10.3 years) and mixed brown race. Associated diseases were relatively few: a single patient was diabetic and 25% had high blood pressure. However, most patients were overweight or obese (mean BMI: 27.87 ± 6.87) and/or had sedentary lifestyles (56%). Most complained of tiredness but not of poor sleep quality. Half were snorers and 21% of the women were menopausal. No patient smoked or consumed alcohol regularly.

The vast majority of the patients had long-standing SLE (60 [11; 168]) and controlled or mild disease activity (0 [0; 4]) on SLEDAI-2k, as shown by the low cumulative dose of corticoids and the small percentage of patients in current use of corticoids (26%). All patients used hydroxychloroquine, 32% used mycophenolate mofetil, and a single patient used methotrexate. No patient was treated with immunobiological. Despite the long duration of SLE in most cases, accumulated sequelae related to SLE or treatment for SLE were minimal, as reflected by the low SLICC-DI scores (Table 1).

### OSA in SLE patients vs. healthy controls

OSA was predominantly mild in SLE patients, and the prevalence was statistically similar for SLE patients (26.4%) and controls (35%) (*p* = 0.360). Likewise, the two groups did not differ significantly regarding ODI, total sleep time spent with SpO_2_ < 90%, and minimum SpO_2_. Moreover, the ESS scores indicated that most SLE patients had no problems with daytime sleepiness (68%) (Table 2).

**Table 2:** HSAT parameters analysis in SLE patients and controls

	**Controls** (*n* = 20)	**SLE** (*n* = 19)	*p***-value**	**Effect size (CI 95% for Cohen’s d)**^**1**^	Statistical Power
**Sex**			1.0	0	5%
Female	18 (90)	17 (89.5)			
Male	2 (10)	2 (10.5)			
**Age** (years)	34.9 ± 9.4	35.9 ± 10.1	0.7	−0.2 (−0.8; 0.4)	9%
**BMI** (kg/m^2^)	28.8 ± 5.9	27.9 ± 6.7	0.6	0.1 (−0.5; 0.7)	6.2%
**BMI** (classification)			0.2	0.2	21.5%
Eutrophic	6 (30)	7 (36.8)			
Overweight	5 (25)	8 (42.1)			
Obese	9 (45)	4 (21.1)			
**Apnea-Hypopnea Index**	3.33 (1.53; 5.6)	2.95 (1.35; 4.75)	0.5	−0.053 (−0.7; 0.6)	5.3%
**Apnea-Hypopnea Index**			0.3	0.1	7,40%
< 5	13 (65)	14 (73.7)			
5 to < 15	6 (30)	4 (21.1)			
≥15	1 (5)	1 (5.3)			
**Time of monitoring (min)**	411 ± 109	470 ± 120	0.1	−0.515 (−1.1; 0.1)	35%
**Minimum SpO**_**2**_ **(%)**	90.6 ± 3.5	90.0 ± 4.7	0.7	0.132 (−0.5; 0.7)	6.8%
**T90%**	0 (0; 0.5)	0 (0; 0)	0.5	−0.128 (−0.7; 0.5)	6.7%
**ODI**	3.2 (1.7; 5.2)	2.8 (1; 4.8)	0.6	−0.096 (−0.7; 0.5)	5.9%
**Epworth sleepiness scale**	-	9 (5; 12)	-	-	-
**Epworth sleepiness scale**			-	-	-
≤10	-	13 (68.4)			
> 10	-	6 (31.6)			

Abbreviations: HSAT – Home sleep apnea testing, SLE – Systemic lupus erythematosus, BMI – body mass index, Minimum SpO_2_ – minimum peripheral oxygen saturation, T90% – total time spent with oxygenation < 90%, ODI – oxygen desaturation index

Categorical variables expressed as absolute numbers and percentages (in parentheses). Quantitative variables expressed as mean value ± standard deviation, or median and interquartile range (in parentheses).

^1^ Cohen’s *d* used for continuous data, and Cohen’s *w* for categorical data.

SLE patients with OSA displayed higher levels of BMI (33.1 ± 9.25 vs. 26 ± 5 kg/m^2^, *p* = 0.043) and greater frequency of obesity (60% vs. 7%, *p* = 0.025) compared to patients without OSA. The remaining variables were statistically similar for the two groups (Table 1).

SLE patients with and without SLE had similar disease activity scores (SLEDAI-2k), SLICC-DI and current corticoid dose (*p* > 0.05). Patients with OSA had a smaller cumulative dose of corticoids over the course of the disease, but the difference was not significant (*p* = 0.07) (Table 1).

### Evaluation of OSA screening tools

The STOP-Bang questionnaire revealed higher levels for patients with OSA than for patients without OSA (3 [2; 3] vs. 1 [0; 2], *p* < 0.001), but the observed levels did not reach the cut-off for high risk of OSA (≥5). A similar pattern was observed with the NoSAS questionnaire (7 [7; 9] vs. 2 [0; 3], *p* = 0.005), but when the NoSAS scores were categorized according to the usual cut-off (≥8), the difference was non-significant (*p* = 0.058) (Table 3).

**Table 3:** Scores obtained with two OSA screening tools (STOP-Bang and NoSAS) in SLE patients

	**SLE** (*n* = 19)	**AHI** (events/hour)		*p***-value**	**Effect size (CI 95% for Cohen’s d)**^**1**^	Statistical Power
		** < 5** (*n* = 14)	**≥ 5** (*n* = 5)			
**STOP-Bang**	1 (0; 2)	1 (0; 2)	3 (2; 3)	**0.0**	**-1.9 (−0.3; −1.0)**	**94%**
**STOP-Bang**				-	-	-
< 5	19 (100)	14 (100)	5 (100)			
≥5	0 (0)	0 (0)	0 (0)			
**NoSAS**	3 (2; 7)	2 (0; 3)	7 (7; 9)	**0.0**	**-2.4 (−0.3; −1.0)**	**98%**
**NoSAS**				0.05	0.3	40%
< 8	17 (89.5)	14 (100)	3 (60)			
≥8	2 (10.5)	0 (0)	2 (40)			

Abbreviations: SLE - Systemic lupus erythematosus, AIH – apnea-hypopnea index

Categorical variables expressed as absolute numbers and percentages (in parentheses). Quantitative variables expressed as mean value ± standard deviation, or median and interquartile range (in parentheses).

^1^ Cohen’s *d* used for continuous data, and Cohen’s *w* for categorical data.

**Fig. 2:**
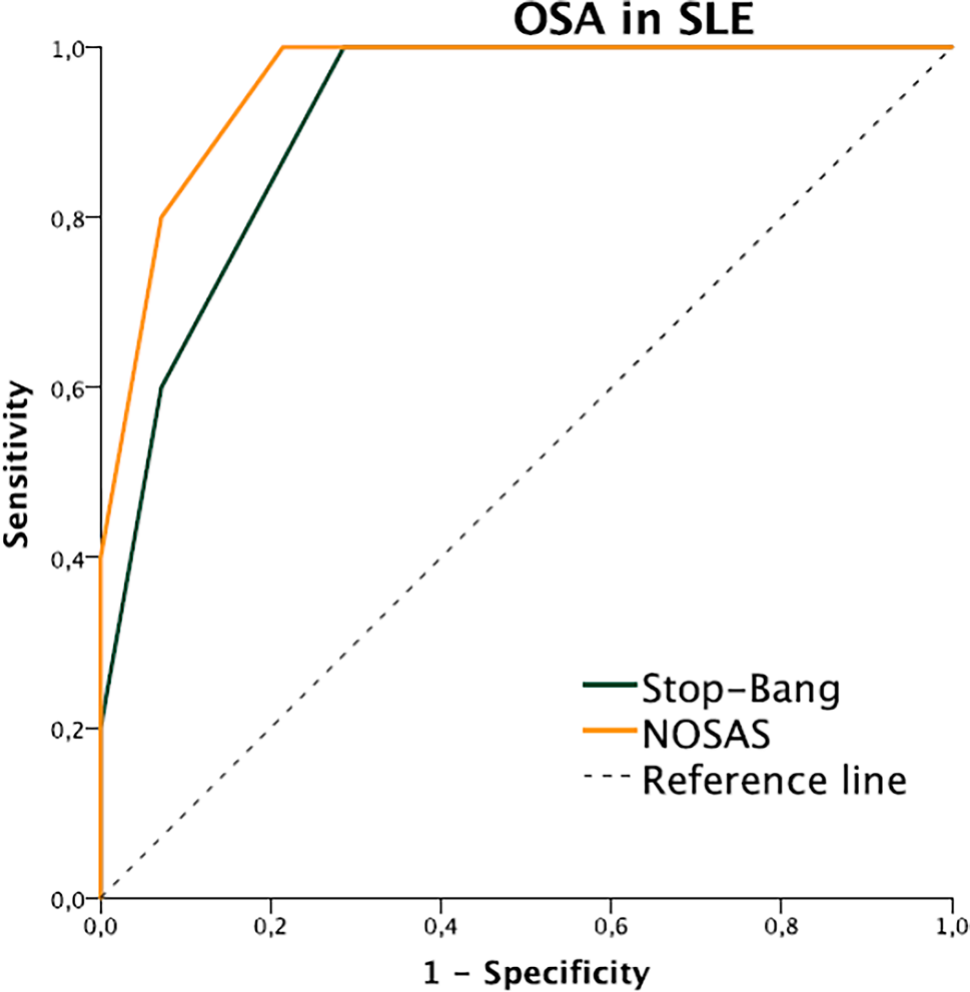
ROC curve evaluating the performance of the OSA screening tools STOP-Bang and NoSAS in SLE patients. OSA: obstructive sleep apnea, SLE: systemic lupus erythematosus

When the scores were submitted to ROC curve analysis, NoSAS yielded higher AUC values (0.957; IC95% 0.710–1.000, *p* = 0.003) than STOP-Bang (0.914; IC95% 0.786–1.000, *p* = 0.003), with 100% sensitivity and 78.6% specificity at the cut-off of ≥4. STOP-Bang was most accurate when a cut-off of ≥ 2 was used, attaining 100% sensitivity and 71.4% specificity. Our results suggest that NoSAS is slightly more efficient at excluding patients without OSA (Table 4; Fig. 2).

**Table 4:** Diagnostic performance of two OSA screening tools (STOP-Bang and NoSAS) in SLE patients

	OSA in SLE patients						
	Cut-off	**AUC-ROC** (95% CI)	**Sensitivity** (%)	**Specificity** (%)	**PPV** (%)	**NPV** (%)	*p***-value**
**STOP-Bang**	≥2	0.914 (0.8; 1.0)	100	71.4	55.1	100	**0.0**
**NoSAS**	≥4	0.957 (0.7; 1.0)	100	78.6	62.1	100	**0.0**

Abbreviations: OSA – Obstructive sleep apnea, SLE - Systemic lupus erythematosus, AUC-ROC = area under the ROC curve; CI = confidence interval; PPV = positive predictive value; NPV = negative predictive value

As for predictive power, PPV was 55.1% and NPV was 100% for STOP-Bang, and 62.1 and 100% for NoSAS, indicating that both tools were excellent at predicting the absence of OSA, while NoSAS was best at predicting the presence of OSA (Table 4).

## Discussion

This is to our knowledge the first study to establish cut-offs for OSA screening tools in SLE patients. Despite it appears to be important comorbidity in SLE, it is underdiagnosed and poorly explored in these patient groups. An inexpensive and readily accessible sleep apnea screening tool would help address this problem.

Few objective evaluations of sleep-related disorders in SLE using polysomnography or HSAT ve been published so far. The literature shows periodic limb movement and OSA to be the most common sleep disorders in SLE patients. In this population, the prevalence of OSA is 26–68% [9, 15–17]. Of note, several factors may explain the discrepancies in OSA prevalence observed across studies: (i) differences in diagnostic methods, as the use of HSAT is less accurate than full PSG; (ii) the fact that most patients in our study had minimal cumulative damage, which may have influenced the observed prevalence of OSA, unlike studies that included patients with higher disease activity; and (iii) geographical location.

We observed a high frequency of OSA in our sample of SLE patients (26.3%), matching the prevalence reported worldwide [6], but the frequency of OSA was not significantly higher in SLE patients than in controls. A similar result was reported by Sahebari M and col., when evaluating sleep disturbances in recently diagnosed SLE patients [16]. However, it has remained a matter of debate for years and other authors have associated SLE with greater prevalence of OSA and more complaints of sleep disturbances compared to healthy controls [9, 15, 17].

These discrepancies may be explained by the method used to diagnose OSA: in this study we used HSAT, which is less accurate than complete polysomnography. The patients were submitted to an HSAT because it is an exam more easily accessible, less complex to perform, and can be conducted in the patient’s home environment [7]. Other explanation may be due to differences between study populations: most of our patients displayed low disease activity on SLEDAI-2k and little cumulative damage on SDI, despite the relatively extended time of disease. Finally, geographical location is known to influence the prevalence of respiratory sleep disorders [6, 16].

The positive association between BMI and OSA observed in our study is consistent with population-based studies demonstrating that obesity is one of the main risk factors for OSA [5, 6]. In fact, obese individuals are about ten times more likely to have clinically significant OSA than normal-weight individuals [5, 6]. OSA is known to be associated with systemic inflammation, hypertension and metabolic disorders [31]. This association may further amplify the cardiovascular morbidity observed in autoimmune diseases, since chronic inflammation is recognized as one of the main factors contributing to the increased cardiovascular risk in patients with rheumatic diseases. This finding underscores the importance of investigating the relationship between SLE and OSA [32, 33] In line with this, our study highligths the need for sleep apnea screening in SLE patients, especially in obese patients, even in the absence of excessive daytime sleepiness.

Interestingly, though more complex and not yet well understood, OSA also appears to trigger or worsen autoimmune and rheumatological disease [10]. Based on the observed SLEDAI2-k and SLICC-DI scores, OSA was not significantly associated with disease activity and accumulated damage, possibly due to the profile of our SLE patients, most of whom displayed low disease activity and low cumulative dose of corticoids.

On the other hand, disease activity was associated with changes in sleep quality and objectively measured sleep efficiency, slow-wave sleep percentage, and sleep fragmentation in a study by Valenci-Flores M. [15]. In another study from 2021 involving 14 recently diagnosed SLE patients with active disease (SLEDAI-2k > 4), sleep disorders were not significantly correlated with disease activity, suggesting that SLE chronicity and its complications are more important for the development of sleep disorders in this patient population [16]. Nevertheless, in a recent study on 42 SLE patients using Watch-PAT, Meidan R and col. reported a positive association between SLEDAI-2k scores and moderate/severe OSA and a positive association between SLICC-DI scores and AHI in the multivariate analysis [9].

The high prevalence of OSA worldwide and the scarcity of sleep clinics (especially in poor regions) justify the development of screening tools to identify patients for polysomnography [7, 13, 26]. Unfortunately, available data suggest that most cases of obstructive sleep apnea remain undiagnosed or inadequately treated. In this study, we evaluated two major and widely employed OSA screening tools validated for our population, but the tools were also chosen because they include risk factors for OSA other than symptoms [6, 13, 29, 30].

Importantly, the scarcity of a reliable screening questionnaire for sleep apnea in SLE raises the issue of which of these patients should undergo formal physiological testing for OSA, either through in-laboratory or home-based testing. NoSAS and STOP-Bang were found to be efficient OSA screening tools in SLE patients. NoSAS yielded a larger area under the ROC curve in SLE patients, but this was achieved at a lower cut-off (≥4) than the cut-off (≥8) described in the original study [30, 31]. AUC-ROC was also large for STOP-Bang, with ≥2 as the most accurate cut-off. Despite the risk of bias associated with the small size of our sample, the excellent AUC-ROC values observed suggest our results are reliable. These findings suggest that, even with a small sample size, the magnitude of the observed effect was large enough to ensure a high probability of detecting real differences between the groups evaluated.

Our findings match those of Balbi GGM and col. [34] who investigated the performance of the same two OSA screening tools in patients with primary antiphospholipid syndrome (PAS). Both tools were useful in screening for OSA in PAS patients, but the area under the ROC curve was larger for NoSAS than for STOP-Bang, although the most accurate cut-off for NoSAS was ≥10, differing from the original study [34]. Based on these considerations, we believe the observed differences in cut-offs are likely related to the profile of our study population: autoimmune disease involving a complex inflammatory cascade with changes in innate immunity mediated by specific cytokines (such as interferon-alpha) and adaptive immune activation, with production of self-reactive antibodies [2, 23]. These traits differ considerably from those of the original studies on NoSAS and STOP-Bang [29, 30].

Our study has some limitations: (i) the small sample size, due to difficulties with HSAT, (ii) single-center design and (iii) use of portable monitoring instead of full polysomnography. This prevented us from performing logistic regressions to identify independent predictors of OSA. However, we evaluated the statistical power of the study, based on the effect size of each association. These analyses provided the magnitude of the differences between groups, even though the small sample size may have limited the ability to detect statistical significance.

## Conclusion

In this study, patients with SLE and controls exhibited a similar frequency of OSA. Both OSA screening tools demonstrated reasonable accuracy in detecting OSA in this population, even at cut-off values lower than those typically applied. Given the underdiagnosis and limited investigation of OSA among SLE patients, the availability of reliable screening questionnaires remains crucial for identifying individuals who should undergo formal physiological testing. Further studies with larger cohorts are warranted to clarify the association between SLE and OSA.

## Data Availability

The datasets used and analyzed during the current study are available from the corresponding author on reasonable request. The data are not publicly available asthey contain confidential information that may compromisse the privacy/consente of the participants.

## Abbreviations

SLE: Uystemic lupus erythematosus
OSA: Obstructive sleep apnea
PSG: Polysomnography
BMI: Body mass index
ACR/EULAR: American College of Rheumatology/the European League Against Rheumatism
ESS: Epworth sleepiness scale
AHI: Apnea-hypopnea index
SLEDAI-2k: Systemic Lupus Erythematous Disease Activity Index
SLICC-DI: Systemic Lupus International Collaborating Clinics/American College of Rheumatology Damage Index
HSAT: Home sleep apnea test
Sp)2: Peripheral oxygen saturation
ODI: Oxygen desaturation index
ROC: Receiver operating characteristic
AUC: Area under the curve
CI: Confidence intervals
STOP-BANG: Snoring, Tired, Observed apneas, Pressure, BMI, Age, Neck size, Gender
